# Supplementary material for: Household food insecurity and associated factors in South Ari district, Southern Ethiopia: A community-based cross-sectional study
Source: PLoS One. 2023 Apr 12;18(4):e0284252. doi: 10.1371/journal.pone.0284252 (PMC10096288; doi:10.1371/journal.pone.0284252)
Supplement: S1 File — (DOCX) [file pone.0284252.s001.docx]

**Informed Written Consent Form**

**Dear Respondent**,

This study is proposed to assess household food insecurity and factors associated with it, and you are chosen to participate in this study. To attain the objective, we are asking for your help. Your answers are completely confidential. It is your full right to refuse to answer any or all of the questions. If you don't want to participate, you can stop the interviewer at any point. However, your honest answers to these questions will help us in better understanding. So; we request your truthful and keen participation.

Would you willing to participate?

_________ Yes, I want to participate in the study

_________ No, I don't participate in the study.

If the answer is No, acknowledge and proceed to the next respondent

Interviewer’s Name and Signature __________________________

Supervisors’ Name and Signature ___________________________

Date of interview______________ Starting time____________ completed time ____________

Name of the Gote ___________

**English version questionnaire**

**Instructions:** The respondent should be the mother/caregivers and are expected to answer issues related to the household.

**Questionnaire code: _______**

**Section A. Socio-demographic and economic characteristics**

| **Name** | **Variables** | **Responses** | | **Skip to Q** |
| --- | --- | --- | --- | --- |
| **A1** | Identification number (ID) |  | |  |
| **A2** | Kebele |  | |  |
| **A3** | Sex of the household head | 1. Male 2. Female | |  |
| **A4** | Age of the household head |  | |  |
| **A5** | Educational status of the household head | 1. No formal education 2. Primary education 3. Secondary education 4. College/University | |  |
| **A6** | Total number of household members | ______________ | |  |
| **A7** | Number of household members whose age is below 15 and above 65 | ___________________________ | |  |
| **A8** | Number of household members whose age is between 15 and 65 | ___________________________ | |  |
| **A9** | Current marital status | **1**. Single  **2**. Married  **3.** Widowed  **4.** Divorced | |  |
| **A10** | Religion | **1.** Orthodox  **2.** Protestant  **3.** Muslim  **4.** Catholic  **5.** Others (specify) ________________ | |  |
| **A11** | Ethnicity | **1**. Ari  **2**. Amhara  **3**. Wolaita  **4**. Goffa  **5.** Other (specify) __________ | |  |
| **Household Assets** | | | | |
| **Now I will ask you about some fixed assets that your household has.** | | | | |
| Does the household have any of the following properties? **(Circle)** | | | **Yes** | **No** |
| **A12** | Functioning radio/Tape recorder/CD player | | 1 | 0 |
| **A13** | Functioning Television | | 1 | 0 |
| **A14** | Gas Stove | | 1 | 0 |
| **A15** | Kerosene stove | | 1 | 0 |
| **A16** | Electric stove | | 1 | 0 |
| **A17** | Bicycle | | 1 | 0 |
| **A18** | Motor Cycle | | 1 | 0 |
| **A19** | Cart/Gari | | 1 | 0 |
| **A20** | Watch (Hand/Wall) | | 1 | 0 |
| **A21** | Mobile phone | | 1 | 0 |
| **A22** | Plough | | 1 | 0 |
| **A23** | Sofa | | 1 | 0 |
| **A24** | Spring mattress | | 1 | 0 |
| **A25** | Sponge/Foam mattress | | 1 | 0 |
| **A26** | Cotton mattress | | 1 | 0 |
| **A27** | Grass Mattress | | 1 | 0 |
| **A28** | Chair/Stool | | 1 | 0 |
| **A29** | Generator | | 1 | 0 |
| **A30** | Milling | | 1 | 0 |
| **A31** | Water pump | | 1 | 0 |
|  | **Does the household have any of the following animals?** | | **1. Yes 0. No** | **How many?** |
| **A32** | Oxen | |  |  |
| **A33** | Cows | |  |  |
| **A34** | Horse/Mules | |  |  |
| **A35** | Goats/Sheep | |  |  |
| **A36** | Chickens | |  |  |
| **A37** | Donkey | |  |  |

**Section B: Household Food Insecurity Access Scale and related questions**

|  | **Question** | **Response** |
| --- | --- | --- |
| **B 1** | In the past four weeks, did you worry that your household would not have enough food | 1= Yes  0 = No |
| **B _1.1_** | If yes, how often did this happen? | 1 = Rarely (1-2times)  2 = Sometimes (3 to 10 times)  3 = Often (more than 10 times) |
| **B 2** | In the past four weeks, were you or any household member not able to eat the kinds of foods you preferred because of a lack of resources? | 1 = Yes  0 = No |
| **B _2.1_** | If yes, how often did this happen? | 1 = Rarely (1-2times)  2 = Sometimes (3 to 10 times)  3 = Often (more than 10 times) |
| **B 3** | In the past four weeks, did you or any household member have to eat a limited variety of foods due to a lack of resources? | 1 = Yes  0 = No |
| **B _3.1_** | If yes, how often did this happen? | 1 = Rarely (1-2times)  2 = Sometimes (3 to 10 times)  3 = Often (more than 10 times) |
| **B 4** | In the past four weeks, did you or any household member have to eat some foods that you really did not want to eat because of a lack of resources to obtain other types of food? | 1 = Yes  0 = No |
| **B _4.1_** | If yes, how often did this happen? | 1 = Rarely (1-2times)  2 = Sometimes (3 to 10 times)  3 = Often (more than 10 times) |
| **B 5** | In the past four weeks, did you or any household member have to eat a smaller meal than you felt you needed because there was not enough food? | 1 = Yes  0 = No |
| **B _5.1_** | If yes, how often did this happen? | 1 = Rarely (1-2times)  2 = Sometimes (3 to 10 times)  3 = Often (more than 10 times) |
| **B 6** | In the past four weeks, did you or any household member have to eat fewer meals in a day because there was not enough food? | 1 = Yes  0 = No |
| **B _6.1_** | If yes, how often did this happen? | 1 = Rarely (1-2times)  2 = Sometimes (3 to 10 times)  3 = Often (more than 10 times) |
| **B 7** | In the past four weeks, was there ever no food to eat of any kind in your household because of a lack of resources to get food? | 1= Yes  0 = No |
| **B _7.1_** | If yes, how often did this happen? | 1 = Rarely (1-2times)  2 = Sometimes (3 to 10 times)  3 = Often (more than 10 times) |
| **B 8** | In the past four weeks, did you or any household member go to sleep at night hungry because there was not enough food? | 1 = Yes  0 = No |
| **B _8.1_** | If yes, how often did this happen? | 1 = Rarely (1-2times)  2 = Sometimes (3 to 10 times)  3 = Often (more than 10 times) |
| **B 9** | In the past four weeks, did you or any household member go a whole day and night without eating anything because there was not enough food? | 1= Yes  0 = No |
| **B _9.1_** | If yes, how often did this happen? | 1 = Rarely (1-2times)  2 = Sometimes (3 to 10 times)  3 = Often (more than 10 times) |
| **Household Food Insecurity Related Questions** | | |
| **B 10** | Did you have your own land for farm? | 1. Yes 0. No |
| **B 11** | If yes how much is the size (hectares) | ________ |
| **B 12** | Did you use agricultural input (fertilizer, improved seed, insecticide, pesticide) | 1. Yes 0. No |
| **B 13** | Did you have access to saving and credit | 1. Yes 0. No |
| **B 14** | Did you use agricultural extension service | 1. Yes 0. No |
| **B 15** | Do you use safety-net service | 1. Yes 0. No |

***Thank you for your participation!!***
