# Supplementary material for: Household food insecurity and associated factors in South Ari district, Southern Ethiopia: A community-based cross-sectional study
Source: PLoS One. 2023 Apr 12;18(4):e0284252. doi: 10.1371/journal.pone.0284252 (PMC10096288; doi:10.1371/journal.pone.0284252)
Supplement: Attachment — Submitted filename: Response to Reviewers.docx [file pone.0284252.s003.docx]

| **Responses to the editor’s comment**  Editor’s comments and suggestions are considered and carefully revised in the manuscript as per the suggestions and comments. I am very thankful to the potential editor for the suggestions and comments, which substantially improved the manuscript. | |
| --- | --- |
| **Comments and questions** | **Responses** |
| 1. Please ensure that your manuscript meets PLOS ONE's style requirements, including those for file naming. The PLOS ONE style templates can be found at <https://journals.plos.org/plosone/s/file?id=wjVg/PLOSOne_formatting_sample_main_body.pdf> and <https://journals.plos.org/plosone/s/file?id=ba62/PLOSOne_formatting_sample_title_authors_affiliations.pdf> | Thank you, dear editor. We have rewritten and revised the manuscript based on PLOS ONE's style requirements. |

| **Responses to the Reviewers' comments and questions**  Reviewer’s comments are considered and carefully revised in the manuscript as per the suggestions and comments. We are very thankful to the potential reviewers for their suggestions and comments, which substantially improved the manuscript. | |
| --- | --- |
| **Reviewer #1 comment and question** | **Responses Reviewer #1** |
| 1. On your Sample size determination, you used a 4% margin of error but the proportion (P) is 75.8%. There are recommendations to use a different value of margin of error. For example, if the proportion is 40-50%, better to use 4 to maximize the sample size. So it is better to use a 5% margin of error if the proportion is greater than or equal to 50%. | Thank you so much, dear reviewer, for your very important suggestions. On the sample size determination,  we used a 4% margin of error (d) to increase the degree of precision required for generalizing the finding since a small value of margin of error indicates high precision. If a 5% margin of error was used, the sample size was 464, which is much lower than 727 (the sample size used in this study) and this might lower the precision of the estimate. As we aim to get a more accurate or precise understanding of a population, then a low margin of error would be better (1). As far as our knowledge, there are also recommendations to use a different value of margin or error depending on proportion: for p close to 50%, d is assumed to be close to 5%; and for a smaller/ larger value of proportion from 50%, d is assumed to be lower than 5% and this value will give a sample size sufficiently large to guarantee an accurate prediction. After considering the feasibility of conducting the study and obtaining a precise estimate, we decide on an optimal sample size (n=727) for our study. |
| 2. In the result section, paragraph 1, line 2 you said that 290 (940.4%). Please edit this grammar error as 94.4%. | Thank you so much, dear reviewer, for pointing out this grammar error. It is corrected as 94.4% and revised on the manuscript accordingly. **(Line 242)** |
| 3. At figure 1: "Schematic presentation of the sampling procedure for the selection of study participants in South Ari District, Southern Ethiopia, 2021." There was a problem during the proportional allocation of a sample at Pella9. Here the population size was 53, however you draw 102 samples. So why? | Dear reviewer, thank you very much for your meticulous observation. It was a grammatical error and we apologize for that. The name of the kebele is Pella and the population size of the kebele was 1,046. You raised a very important point and we revised it accordingly (**Figure** **1**) |
| **Reviewer #2 comment and question** | **Responses to Reviewer #2** |
| **Introduction**   - An introduction is too long with redundancy, so try to make it smart and keep the right flow. | Thank you so much, dear reviewer. We revised it as per the suggestion. |
| **Discussion**   - It is advisable to explain the study objective at the beginning and start the discussion with a summary of your study with the main findings. | Dear reviewer, Thank you very much for your important comments and revised the manuscript accordingly. We included one paragraph at the beginning of the discussion section, which summarizes the objective of the study followed by the main findings of this study “This study assessed household food insecurity and associated factors in South Ari district, Southern Ethiopia. Accordingly, 44% of the households in the South Ari District were food insecure. Larger family size, high dependency ratio, poor household wealth index, not using agricultural extension service, and non-beneficiary of productive safety net program were risk factors associated with household food insecurity” **(Line 318-322).** |
| - Better to mention the reference for most of your possible explanations (some scientific facts | Thank you so much, dear reviewer, for your meticulous observation. We included evidence supporting possible explanations and references in the revised manuscript. |

**References**

1.	Fairbairn W, Kessler A. Practical Advice for Selecting Sample Sizes. 2021.
