# Supplementary material for: Household food insecurity and associated factors in South Ari district, Southern Ethiopia: A community-based cross-sectional study
Source: PLoS One. 2023 Apr 12;18(4):e0284252. doi: 10.1371/journal.pone.0284252 (PMC10096288; doi:10.1371/journal.pone.0284252)
Supplement: Attachment — Submitted filename: Response to Reviewers.docx [file pone.0284252.s004.docx]

**A point-by-point response to Editor and Reviewers**

**Responses to the editor’s and Reviewers' comments and questions**

We thank the Editor and reviewers for their thorough reading of the manuscript and insightful comments. Based on the report we received, we have addressed the important comments and suggestions given by the reviewers in the revised submission, and we believe that the comments substantially improved the manuscript. An unmarked version of the revised paper without tracked changes was uploaded as a separate file labeled “Manuscript”. A marked-up copy of the manuscript that highlights changes made to the original version was uploaded as a separate file labeled “Revised Manuscript with Track Changes”. Below are the point-by-point responses to the reviewers’ comments.

| **Reviewer #2 comments** | **Responses to Reviewer #2** |
| --- | --- |
| The main strength of this study is they explored household food insecurity and associated factors in Ethiopia, where this issue is important for beneficiaries and the existing sciences, so I would like to appreciate the authors once again.  As per my previous review, the authors addressed all their given comments and suggestions in this revised manuscript. | Thank you very much dear reviewer for taking the time for reading the manuscript and giving very insightful comments and suggestions. We believe that your comments and suggestions substantially improved the manuscript. |
| **Reviewer #3 comments** | **Responses to Reviewer #3** |
| The authors attempted to assess food insecurity in some parts of Ethiopia. Sub-Saharan countries are highly challenged by food security. Such types of research are highly encouraged to see the different perspectives of challenges behind food insecurity. | Dear reviewer thank you so much for giving these encouraging words. The prevalence of household food insecurity is very high from time to time due to different reasons in Sub-Saharan countries, especially during the COVID-19 pandemic. Hence, understanding food insecurity and responsible factors from a different perspective is very crucial for tackling its consequences in the region. |
| Primarily it is better if the title “food insecurity” is covered by the Economic perspective of studies or the Agricultural field of studies. | Thank you so much dear reviewer for raising this point. Addressing food insecurity from an economic and agricultural perspective is highly important. However, household food insecurity is one of the major public health problems in African countries, especially in Sub-Saharan countries. The consequences of food insecurity are not limited to economic or agricultural perspectives. It is anticipated that household food insecurity can negatively affect children’s and women’s food consumption, including reduced quantity and quality of diet varieties and nutrient intakes, and people’s health in different ways. According to the United Nations International Children’s Emergency Fund (UNICEF) conceptual framework, food insecurity is one of the proximate determinants of poor child nutritional status. Adequate nutrition is crucial for health and development. However, the presence of food insecurity at the household level indicates a high level of vulnerability to extensive consequences, including psychosocial dysfunction among household members, especially children, socioeconomic predicaments, and poor overall health status. Malnutrition is one of the most severe consequences of food insecurity. However, there is limited evidence in assessing food insecurity and responsible factors from a public health point of view. Therefore, covering household food insecurity from a different perspective (economic, agricultural, and public health) collaboratively is very crucial for tackling various consequences of food insecurity. |
| Since the title is “food insecurity”, in the introduction section of the paper, the authors failed to stipulate the impact of food insecurity. Whereas the introduction grossly stated malnutrition (the immediate and one impact of food insecurity). This is understandable since malnutrition is a burning public health issue. But deviate from the initial title of the study. A better solution could be modifying the title to malnutrition or rewriting introduction section. | Thank you dear reviewer for your meticulous suggestion. Our study aimed to assess household food insecurity and associated factors. So, we restructured and incorporated the consequences or impact of household food insecurity from different perspectives in the revised manuscript.  **Line 72-96, 1276-135.** |
| In the methodology section, the authors did not put who the respondents are. If the answer to the above question is “household’s heads” did the authors find them whenever possible? | Dear reviewer, thank you so much for this interesting point. The respondents for this study were household heads.  **Line 153,154, 177.**  The author visited at least three times to randomly selected households to find household heads for getting appropriate data regarding the study. If the household heads did not found after the third visit, then the immediate next household will be included, however, we practically did not face this kind of challenge. We found the household’s heads on at least three visits. |
| What do you do for those seriously ill patients as an ethical issue? | Dear reviewer, thank you very much for the comment and concern on the ethical issue. Those with household heads who were unable to communicate and give correct data to the interview during data collection due to serious illness and mental problems were excluded. The required data were collected through face-to-face interviews with study participants able to provide the data. So, this might take some minutes for each participant. Those households with food insecurity were linked to the agricultural and rural development office for intervention aiming to improve food security status. But, the study had no direct and immediate benefit for participating households. Seriously ill patients/participants unable to provide the required data were excluded from the study, however, in the future, the evidence generated from the study was generalized for the study setting and might help to improve food security in the setting as a whole. **Line 154-155, 246.** |
| In the result section where is the direct public health impact of food insecurity? As if the authors stated the consequences of food insecurity (for example, malnutrition) but you did not show the extent of malnutrition. Among those food insecure households, how many of them have malnutrition persons? | Thank you so much dear reviewer for your meticulous observation. Household food insecurity by itself is one of the major public health problems in Sub-Saharan countries. Herein, our study primarily aimed and assessed household food insecurity and responsible factors for it in South Ari District, Southern Ethiopia. Hence, here in the result section, the author described the prevalence of household food insecurity, related characteristics, and the factors responsible for it. The consequences of food insecurity are various. It is anticipated that household food insecurity can negatively affect children’s and women’s food consumption, including reduced quantity and quality of diet varieties and nutrient intakes, and people’s health in different ways. Food insecurity is one of the proximate determinants of poor child nutritional status /malnutrition/. However, the primary aim of this study was not to assess the consequences or impact of household food insecurity like malnutrition. The authors were primarily interested in the prevalence of household food insecurity in the district and the responsible factors for it. That is why we did not show the prevalence of malnutrition among food-insecure and food-secure households. But, implementing appropriate interventions by addressing factors responsible for food insecurity is not only beneficial for improving food security status but also important for tackling the impact of food insecurity. |
| The paper failed to write a proper conclusion section and it is erratic. Of course, it is hard to put precise public health conclusions based on the title selected employed. | Dear reviewer, thank you very much for your valuable comment. We revised the conclusion in the revised manuscript based on your suggestions. **Line 383-395** |
